# Integrating virtual reality into multidisciplinary care for Parkinson's disease: A narrative review

**DOI:** 10.1177/1877718X251323916

**Published:** 2025-03-16

**Authors:** Daniela Pimenta Silva, Filipa Pona-Ferreira, Ricardo Cacho, Beatriz Santos, Teresa Lobo, Raquel Bouça-Machado, Joaquim J Ferreira

**Affiliations:** 1 Centro de Estudos Egas Moniz, Faculdade de Medicina, Universidade de Lisboa, Lisbon, Portugal; 2 CNS-Campus Neurológico, Torres Vedras, Portugal; 3 Laboratory of Clinical Pharmacology and Therapeutics, Faculdade de Medicina, Universidade de Lisboa, Lisbon, Portugal

**Keywords:** Parkinson's disease, physiotherapy, clinical exercise, exergaming, virtual reality

## Abstract

Parkinson's disease (PD) is a neurodegenerative disorder characterized by both motor and non-motor symptoms, significantly impacting patients’ functionality and quality of life. Clinical exercise, as part of a multidisciplinary approach, is gaining a crucial role in PD management. Goal-based exercises, combining physical activity with cognitive tasks, external feedback and cues, motor sequencing strategies and dual-tasking may enhance motor learning processes and guide physiotherapy programs.

Virtual reality (VR) and exergaming have also emerged as promising tools in PD rehabilitation, offering challenging activities in multisensory environments. They provide intensive and repetitive training, augmented feedback, and tailored exercises in highly interactive and enriched environments. Clinical studies have presented promising results in people with PD, supported by neuroimaging studies showing distinct brain activation patterns post-VR training. However, heterogeneity in study design and lack of standardized characterization of VR systems hinder further application in PD rehabilitation.

In this review, we appraise the distinguishing features between different VR systems, highlight VR-related motor and cognitive training in PD and explore how VR interventions are aligned with principles of neuroplasticity and motor learning in PD.

## Plain language summary

Parkinson's disease (PD) is a brain disorder characterized by motor and non-motor symptoms, significantly impacting patients’ quality of life. Exercise, along with other treatments, is a crucial element in the management of the disease. Specific exercises that focus on daily activities and that incorporate cognitive tasks and multitasking are the basis of rehabilitation programs. Virtual reality (VR) and exergaming are methods that offer challenging activities in immersive environments, providing intense and repetitive training, with important feedback and personalized exercises in a enticing setting. Although studies have shown positive results, there are still no recommendations for their implementation in clinical practice. This review summarizes VR applications in PD rehabilitation, exploring less known aspects of these interventions and hopefully providing guidance for future research and contributing to find the appropriate place of VR in physiotherapy.

## Introduction

Parkinson's disease (PD) is a slow progressive neurodegenerative disorder with an increasing incidence and prevalence worldwide.^1,2^ It is a complex disease characterized by the development of motor and nonmotor symptoms that significantly impact patients’ functionality and health-related quality of life, as well as caregivers’ burden.^2^ To date, there is no medication to prevent or halt disease progression, with symptomatic relief and disability reduction being the aim of therapeutic approaches. Dopamine replacement therapies remain the most effective treatment to improve motor symptoms,^3,4^ but pharmacological management alone fails to address all disease dimensions. Mounting evidence demonstrate that multidisciplinary care is essential to better manage the complexity of disease manifestations and to achieve an individualized approach.^2,5–8^

Physiotherapy is an important part of this multidisciplinary strategy^2^ with different therapeutic modalities proving beneficial for people with PD (pwPD).^9,10^ While there is no sufficient evidence to recommend one over the other,^9^ current guidelines propose to engage patients in person-specific, enjoyable and feasible exercise regimens, while meeting SMART goals (specific, measurable, attainable, relevant and time-based).^10^ Alternatives to conventional physiotherapy are being increasingly investigated, including martial arts, dance, boxing, Nordic walking and various forms of exergaming.^9^ Adding to the enjoyable and motivating environment, these complementary exercise strategies demand complex motor sequences incorporating attentional tasks and external cueing,^11^ thus providing additional elements to promote motor learning. In particular, virtual reality (VR) and exergaming allow users to engage in challenging functional activities in multisensory environments with constant feedback about movement and performance.

VR has been studied in several rehabilitation contexts, including in PD, with promising albeit inconsistent results.^12–16^ The lack of standardized characterization and categorization of VR systems contributes to the ambiguity surrounding their effectiveness.^17,18^ Evidence has showed that the distinction between specific and nonspecific VR systems may be important to guide physiotherapy and exercise interventions using VR.^17^ Moreover, since different technologies may influence the type of human-computer interaction,^19,20^ questions arise regarding the influence of different VR types, the appropriate clinical settings, the exercises that pwPD most benefit from VR interventions, as well as the best training protocols. Finally, studies have included PD patients with mild to moderate disease, hence the utility of VR in advanced stages and with cognitive impairment still needs to be addressed.

In this narrative review, we aim to explore these unanswered questions. We start by providing an overview on the well-studied exercise and rehabilitation principles in PD, followed by summarizing VR-related features and its role in the context of rehabilitation. Then, we appraise the current literature focusing on the relevance of categorizing VR into specific and nonspecific systems in PD rehabilitation and the presence of the exercise and rehabilitation principles within VR interventions. We hope that by clarifying these relevant questions we can guide future research.

## Clinical exercise and virtual reality – an asset to neurorehabilitation

Clinical exercise and its benefits in PD have been studied through high-quality research studies.^9,21^ The most recent Cochrane review on physiotherapy in the management of PD included 156 randomized control trials (RCT) indicating that various modalities of physical exercise benefit pwPD by improving motor symptoms, quality of life and functional mobility.^21^ With no clear evidence that one modality is superior to others, the authors suggest that fundamental exercise principles should be applied alongside personal preferences.^21^ In addition, exercise prescription in PD have been guided by knowledge from the sports science field^22^ and principles of experience-induced neuroplasticity.^11^ Exercises that are intense, specific to patients’ goals, repetitive, progressive in difficulty and complexity, and variable are major assets in physiotherapy programs for PD.

Understanding motor learning and its influencing factors has been pivotal in further shaping clinical exercise and rehabilitation programs. The loss of dopamine in the posterior striatum leads to dysfunctional corticostriatal networks involved in automatic motor control, thus increasing the recruitment of alternate brain regions linked to cortical reward-based learning.^11,23,24^ Consequently, pwPD rely more on external sources of feedback and may require more time and repetitions to learn a motor skill than age-related healthy subjects.^23,25,26^ External cues, including visual, auditory, and proprioceptive stimuli, compensate the lack of internal generation of movement,^27^ stimulating goal-based learning and directing attention to the task.^28,29^ Basal ganglia dysfunction further impairs motor learning by compromising cognitive function, specifically attentional and executive domains, such as working memory, planning, set shifting and multitasking, which in turn impacts gait and balance in PD, even in the absence of explicit cognitive impairment.^25^ Hence, implementing challenging goal-based exercise strategies not only enhances motor function, but also increases the cognitive engagement, motivation and reward.^10,30^ Reward and motivation are crucial factors in the motor learning process, that also contribute to patient compliance with intervention protocols.^24^

Virtual reality (VR) can be broadly defined as the ‘creation of visual simulations by a computer software which interact in real time with the user, through the synchronization of multiple sensory channels capable of detecting motion in three-dimensional space and simultaneously delivering visual, auditory and/or sensorimotor feedback’.^19,20,31^ It is a versatile technology that enables the creation of countless scenarios that can be adapted and manipulated according to the context being used. In the rehabilitation context this is particularly useful since it allows to effortlessly provide highly intensive and repetitive training protocols while eliciting naturalistic behavioral responses in a safe environment. Simultaneously, VR can demand planning, attention and high-order processing of sensory integration due to its multisensorial augmented feedback.^32^

However, the tendency to group different technologies together and conclude on ‘VR’ effectiveness may contribute to discrepancies in systematic reviews and comparative studies, thus precluding recommendations.^17,20^ Evidence from stroke rehabilitation has demonstrated that custom-made VR systems have more substantial impact on function and activity because principles of motor learning and neuroplasticity are more frequently applied than with commercially available VR systems (e.g., Nintendo Wii or Kinect Xbox).^17,33^ Notably, six neurorehabilitation principles were identified in more than 50% of specifically designed VR systems, including task-specific practice, variable practice, implicit feedback (knowledge about performance), explicit feedback (knowledge about result), increasing difficulty, and repetitive use of the affected limb. Results from this study hold significant implications for VR research.

## Virtual reality interventions in Parkinson's disease

### Search strategy

A PubMed search was performed using the following search terms and their variations: “virtual reality”, “exergaming”, “serious games”, “augmented”, “user-computer interface”, “computer”, “software”, “immersion”, “exercise”, “neurorehabilitation” and “Parkinson disease”. We also searched the reference list of articles identified and selected those we judged relevant. Experimental and quasi-experimental studies were qualitatively analyzed for characterization of VR systems and for the presence of the above-mentioned exercise and neurorehabilitation principles. We further extracted data to characterize intervention settings, types of exercises and training protocols delivered by VR, participants characteristics regarding disease severity and cognitive impairment. Table 1 summarizes the main results.

**Table 1.** Characterization of the VR systems, VR interventions protocols and settings, and inclusion criteria regarding disease severity and cognition in experimental and quasi-experimental studies for the rehabilitation of Parkinson's disease.

	Inclusion criteria (disease severity and cognition)	VR system	Setting, Supervision	Type of exercises / training protocol	Comparator(s)	Exercise and rehabilitation principles	Additional features
*Specific VR systems*							
Mirelman et al., 2011^78^ Open-label study	PD HY II-III, No dementia	Virtual environment with real-life challenges Non-IVR	In-clinic, Supervised by therapist and safety harness	Treadmill training with obstacle negotiation tasks 45 min, 18 sessions, 6weeks	Before-after comparison	Task specificity Progression in intensity and difficulty Dual task Implicit feedback Decision making Attention Response selection	None
Yen et al., 2011^80^ RCT	PD HY II-III, MMSE > 24	Virtools 3.5 with a dynamic balance board Non-IVR	In-clinic, Supervised by therapist	Weight shifting for targeting and weight shifting in daily environments 30 min, 12 sessions, 6 weeks	- Equivalent balance training - No exercise	Task specificity Progression in complexity and difficulty Multisensory stimuli Implicit feedback	None
van den Heuvel et al., 2014^49^ RCT	PD HY II-III, MMSE ≥ 24	Motek Medical Non-IVR	In-clinic, Partially supervised by therapist	Dynamic balance exercises exploring limits of stability, weight shifting, sit-to-stand and dual task exercises 60 min, 10 sessions, 5 weeks	Equivalent conventional balance training	Progression in intensity and difficulty Visual feedback Explicit feedback Dual task	None
Zimmermann et al., 2014^60^ RCT	PD with mild cognitive impairment	CogniPlus vs. Nintendo Wii Non-IVR	In-clinic, Supervised by therapist	Cognitive training: focused attention, working memory, planning, action skills, response inhibition	Nintendo Wii (4 sports games in a seated position)	Task-specific Adapted difficulty (lack of motivation)	None
Palacios-Navarro et al., 2015^45^ Feasibility study	PD without history of falls, MMSE ≥ 24	Customized gaming protocol Non-IVR	In-clinic, Supervised by therapist or caregiver	Lateral leg movements while standing 30 min, 20 sessions, 5 weeks	Before-after comparison	Adapted difficulty Explicit feedback Repetitive practice Motivation (Lack of implicit feedback and visual and auditory cueing)	Ability to store performance data
Mirelman et al., 2016^56^ RCT (V-TIME study)	Older adults (aged 60–90 years) with high risk of falls, including PD HY II-III and no cognitive impairment	Virtual environment with real-life challenges Non-IVR	In-clinic, Supervised by therapist and safety harness	Treadmill training with obstacle negotiation tasks 45 min, 18 sessions, 6 weeks	Equivalent treadmill training protocol without VR	Task specificity Progression in difficulty and complexity Multisensory stimuli Implicit feedback Explicit feedback Attention Planning Dual task Response selection	Additional costs from VR were minimal
Shih et al., 2016^44^ RCT	PD HY I-III, MMSE ≥ 24	Customized balance-based exergaming Non-IVR	In-clinic, Supervised	Reaching tasks with stationary and moving objects: weight shifting, single limb support, arm and leg coordination, stepping, functional transitions, advance motor planning and strategy 50 min, 16 sessions, 8 weeks	Equivalent exercises with reaching activities, weight shifting and marching activities	Task specificity Implicit feedback Repetition Intensity Progression in difficulty and complexity Varied practice Proprioceptive feedback Dual task Planning	Low-cost
Yang et al., 2016^81^ RCT	PD HY II-III, MMSE > 24	Customized VR balance training system Non-IVR	Home-based, Supervised by therapist	Static posture and dynamic weight shifting with simulated daily activities, indoor and outdoor 50 min, 12 sessions, 6 weeks	Equivalent conventional balance training at home	Task specificity Progression in difficulty Visual and auditory feedback Implicit and explicit feedback Repetition Motivation	None
Albiol-Pérez et al., 2017^58^ Open label	PD with gait instability, mild to moderate cognitive impairment	Customized Active Balance Rehabilitation (ARAB) system Non-IVR	In-clinic, Supervised by therapist	Weight transferences in standing and sitting position followed (30 min) by traditional rehabilitation (30 min) 60 min, 15 sessions, 5 weeks	Before-after comparison	Task specificity Adjusted difficulty Auditory feedback Attention Enjoyment	Affordable, portable, and accessible; ability to store performance data
Allen et al., 2017^53^ RCT	PD, MMSE ≥ 24	Customized exergame system Non-IVR	Home-based, unsupervised, 3 home visits to ensure correct use and monitor progression + regular phone calls	Coordinated movements of the arm and hand: correct timing of responses and rapid movement 36 sessions, 12 weeks	‘Usual care’	Implicit feedback Progression in difficulty Enjoyment (Lack of task specificity and variety)	Constraints setting up the equipment at home
Song et al., 2017^46^ RCT	PD, able to walk unaided for ≥ 30 meters, MMSE ≥ 24	StepMania and custom-made step mat Non-IVR	Home-based, Unsupervised, 3 home visits to ensure correct use, safety and monitor progression + regular phone calls	Step training with a cognitive load ≥ 15 min, 36 sessions, 12 weeks	No exercise	Progression in difficulty Implicit and explicit feedback Divided attention Executive function	Custom-made step mat also used for assessment
Maggio et al., 2018^50^ RCT	PD HY < III, mild to moderate cognitive impairment (MMSE 11–26)	Nirvana BTS Semi-IVR	In-clinic, Supervised by therapist	Cognitive training in different domains: executive and visuospatial, attention, speech abilities and memory 60 min, 24 sessions, 8 weeks	Equivalent exercises of conventional cognitive rehabilitation	Multisensory stimuli Implicit and explicit feedback Progression in difficulty Motivation	None
Nuic et al., 2018^52^ Feasibility study	PD with disabling freezing of gait and/or falls, MMSE ≥ 24	‘Toap Run’ videogame Non-IVR	In-clinic, Supervised by therapist	Large amplitude and rapid movements of all four limbs, pelvis and trunk, with lateral, vertical and forward displacements of the legs 20–40 min, 18 sessions, 6 weeks	Before-after comparison	Progression in difficulty and complexity Repetition Attentional demand Visual and auditory cues Motivation	None
Calabro et al., 2019^51^ Preliminary study	PD HY ≤ 3, MMSE ≥ 24	CAREN	In-clinic, Supervised by therapist and safety harness	Conventional PT for 5 weeks (50 min), followed by 3 months of rest and 5 weeks of treadmill training with cognitive challenges with VR (40 min)	Before-after comparison	Task-specific Multisensory stimuli Progression in difficulty Adaptability	Ability to assess patients while training, record performance, custom-made experience High cost
Cikajlo et al., 2019^34^ RCT	PD HY II-III, MMSE ≥ 25	’10Cubes’ exergaming system with Leap Motion Controller	In-clinic, Supervised by therapist	Picking multicolored virtual cubes and placing them with a hand one by one in the box 30 min, 10 sessions,3 weeks	IVR vs. non-IVR (similar protocols)	Task specificity Motivation, enjoyment and interest	Leap Motion Controller also used for assessment
Van der Kolk et al., 2019^55^ RCT	PD HY ≤ 2, MMSE ≥ 24	Not specified Non-IVR	Home-based, remotely supervised through a motivational app and regular phone calls	Aerobic training in a stationary bicycle enhanced by a virtual environment and virtual competitors 30–45 min, 3 times / week, 6 months	Non aerobic training at home: stretching, flexibility, and relaxation exercises. The same strategy with motivational app and phone calls was used.	Progression in intensity Visual feedback Implicit feedback Motivation and reward	Ability to record sessions and to connect to a motivational app to provide feedback on performance
Brandin de La Cruz et al., 2020^48^ Feasibility study	PD, able to walk ≥ 10 meters, no restrictions regarding cognition	Motigravity IVR	In-clinic, Supervised by therapist	Antigravity treadmill combined with IVR for gait rehabilitation with dual-task component 30 min, 12 sessions, 4 weeks	Before-after comparison	Task specificity Dual task	None
Pazzaglia et al., 2020^47^ RCT	PD with low risk of falling and able to perform motor rehabilitation independently MMSE > 25	Nirvana BTS Semi-IVR	In-clinic, Supervised by therapist	Multiple exercises with whole body movements and complex set of sensorimotor control systems 40 min, 18 sessions, 6 weeks	Conventional PT	Multisensory stimuli Visual and auditory feedback	Ability to record sessions
Weijer et al., 2020^67^ Feasibility study	PD HY ≤ 3, Mild cognitive impairment (level 1 from MDS criteria)	AquaSnap™	Home-based, Unsupervised	Attention, working memory, episodic memory, psychomotor speed, and executive function 30 min, 36 sessions, 12 weeks	‘Wait list control’	Progression in difficulty Varied Individualization Reward	None
Maranesi et al., 2022^54^ RCT	PD HY I-III, MMSE ≥ 24	Tymo®, a wireless static and dynamic platform Non-IVR	In-clinic, Supervised by therapist	Patient's body becomes the joystick, moving in space to reach different targets of the game (30 min), followed by conventional PT (20 min) 50 min, 10 sessions, 5 weeks	Traditional rehabilitation: breathing and relaxation, task-oriented exercises, walking with cues, stretching, static and dynamic balance training, flexibility, uni and contralateral coordination	Goal-oriented practice Personalized complexity and intensity Implicit and explicit feedback Dual task	None
*Commercial gaming devices*							
Pompeu et al., 2012^39^ RCT	PD HY I-II, MMSE ≥ 24	Nintendo Wii (Wii Fit games) Non-IVR	In-clinic, Supervised by therapist	Global exercises (30 min), followed by VR: static and dynamic balance, stationary gait, and cognitive demands (response inhibition, decision-making, strategy changes, working memory, divided attention) (30 min) 60 min, 14 sessions, 7 weeks	Global exercises (30 min), followed by equivalent balance training without VR (30 min)	Repetition Multisensory stimuli Visual and auditory cues Implicit and explicit feedback Attention Working memory Decision-making Motivation	None
Herz et al., 2013^69^ Open-label	PD HY II, MMSE ≥ 24	Nintendo Wii (Wii Fit games) Non-IVR	Not specified	Full body motion, UL balance and aerobic exercise 60 min, 12 sessions, 4 weeks	Before-after comparison	Visual cues Adjusted difficulty Motivation	Cost-effective
Pompeu et al., 2014^76^ Feasibility study	PD HY II-III, MMSE ≥ 20	Xbox Kinect (Kinect Adventures) Non-IVR	In-clinic, Supervised by therapist	Motor demands: balance reactions and postural adjustments with fast movement of 4 limbs; cognitive demands: visuospatial attention, attention shifting, decision-making, reaction time, planning 60 min, 14 sessions,5 weeks	Before-after comparison	Visual and auditory cues Dual task Attention Planning Decision-making	None
Liao et al., 2015^72^ RCT	PD HY I-III, MMSE ≥ 24	Nintendo Wii (Wii Fit games and balance board) Non-IVR	In-clinic, Supervised by therapist	10 min yoga exercises, 15 min strengthening, 20 min of balance games, followed by 15 min treadmill training 60 min, 12 sessions, 6 weeks	- Equivalent traditional exercise (45 min), followed by treadmill training (15 min) - Fall-prevention education	Progression in intensity, repetitions and difficulty Visual and auditory feedback Implicit and explicit feedback Attention Problem solving	None
Gandolfi et al., 2017^41^ RCT	PD HY 2.5-3, MMSE ≥ 24	Nintendo Wii (Wii Fit games with balance board) Non-IVR	Home-based, Remotely supervised, presence of caregiver required	Weight shifting, symmetric foot stepping, and controlled movements near the limits of stability 50 min, 21 sessions, 7 weeks	In-clinic (supervised) SIBT: stretching, self-destabilization, external destabilization, and combined self-destabilization and external destabilization exercises	Repetition Complexity Implicit feedback Visual and auditory cues Planning Attention Dual task Motivation	Total cost of rehabilitation using TeleWii was lower than control
Ribas et al., 2017^40^ RCT	PD HY I-III, MMSE ≥ 24	Nintendo Wii (Wii Fit games with balance board) Non-IVR	In-clinic, Supervised by therapist	Motor demands: multidirectional shifts and stationary control of center of gravity, alternating steps; cognitive demands: attention, rapid responses to stimuli, decision-making and response inhibition 30 min, 24 sessions, 12 weeks	Equivalent conventional exercises: warming, stretching, resistance and diagonal exercises for the trunk, neck and limbs	Visual and auditory feedback Implicit feedback Attention Decision-making Motivation	None
Alves et al., 2018^35^ Quasi-experimental	PD HY I-III, MMSE ≥ 24	Nintendo and Xbox Kinect Non-IVR	In-clinic, Supervised by therapist	Motor demands: fast stationary walk avoiding obstacles, alternating steps with game rhythm, alternating steps while moving arms, weight shifting; cognitive demands: response inhibition and planning, sustained attention and visuospatial capacity, divided attention, decision-making 45–60 min, 10 sessions, 5 weeks	- Nintendo Wii vs. Xbox Kinect (similar protocols) - No exercise	Progression in difficulty Visual and auditory cues Attention Planning Decision-making	Inexpensive
deMelo et al., 2018^71^ RCT	PD HY I-III, no restrictions regarding cognition	Xbox Kinect Non-IVR	In-clinic, Supervised by therapist	Motor coordination and physical fitness: stationary gait, involving symmetry and alternating actions and rhythm 20 min, 12 sessions, 4 weeks	- Treadmill training - Functional gait training, with obstacles and visual cues	Intensity Progression in difficulty Implicit feedback Complexity Repetition Multisensory feedback Problem solving	None
Ferraz et al., 2018^70^ RCT	PD HY II-III, no restrictions regarding cognition	Xbox Kinect Non-IVR	In-clinic, Supervised by therapist	Full-body motion with exercises involving strength and muscular endurance, cardiorespiratory fitness, postural balance and executive function 50 min, 24 sessions, 8 weeks	- Bicycle training: aerobic training - Functional gait training: gait with obstacles, stairs, sitting and standing, side gears, balance, activities with balls, step and foot tip exercises, reaching, gait training	Progression in intensity and difficulty Multisensory stimuli Executive function	None
Tollár et al., 2018^75^ RCT	PD HY II-III, MMSE ≥ 24	Xbox Kinect Non-IVR	In-clinic, Supervised by therapist in groups of 4 to 8	Postural control, gait mobility, gait stability, turning, and dynamic and static 60 min, 25 sessions, 5 weeks	- Cycling: aerobic training, without visual cues - No exercise	Intensity Multisensory stimuli Personalized Motivation	None
Tollar et al., 2018^38^ RCT	PD, MMSE ≥ 24	Xbox Kinect Non-IVR	In-clinic, Supervised by therapist in groups of 4 to 8	Sensorimotor and visuomotor agility training (20 min), followed by exergame: dynamic and static balance, coordination, reactions to external and internal perturbations, dual tasking, and the ability to shift between body positions, postures, and tasks (20 min) 60 min, 15 sessions, 3 weeks	No exercise	Task specificity Intensity Variation Progression in difficulty and complexity Auditory and visual cues Implicit feedback Dual task	None
Santos et al., 2019^74^ RCT	PD HY I-III, MMSE ≥ 24	Nintendo Wii (Wii Fit games with balance board) Non-IVR	In-clinic, Supervised by therapist	Lateralization, rotation and extension of the trunk, mobility of the UL, weight transfer, equilibrium reactions and stationary gait 50 min, 16 sessions, 8 weeks	- Conventional exercises only: diagonal, active-assisted and resisted active movements, and walking - VR (20 min), followed by conventional exercises (20 min) (similar protocols)	Specificity	None
Hajebrahimi et al., 2022^42^ RCT	PD HY I-III, No dementia	Nintendo Wii (Wii Fit Plus games, with balance board) Non-IVR	In-clinic, Supervised by therapist	Yoga (stretching exercises), strengthening games and balance games 60 min, 12 sessions, 4 weeks	Equivalent balance and gait training basically focusing on lower extremity movements	Progression in intensity and repetitions Visual and auditory feedback Implicit feedback Cognitive engagement1 Motivation	None
Kashif et al., 2022^37^ RCT	PD HY I-III, MMSE ≥ 24	Nintendo Wii (Wii Fit games, with balance board) Non-IVR	In-clinic, Supervised by therapist	Routine PT (40 min), followed by VR for full-body motion, dynamic and static balance training and motor function (10–15 min), followed by motor imagery 60 min, 12 sessions, 6 weeks	Routine PT: warm-up, stretching, strengthening, relaxation and limb coordination exercises, and core, neck and gait training (40 min), followed by cycling or walking (20 min)	Repetition Implicit feedback Multisensory stimuli	None
*Not specified VR systems*							
Feng et al., 2019^73^ RCT	PD HY 2.5-4, no restrictions regarding cognition	Not specified	Inpatient clinic, Supervised by therapist	Full range of motion, strength, shifting the center of gravity, single leg weight, coordination, quick response, limb flexibility, exercise adaptation, body turning 45 min, 60 sessions, 12 weeks	PT: balance, coordination, physical condition	Task-oriented training Visual feedback Implicit and explicit feedback Motivation	None

RCT: randomized controlled trial; VR: virtual reality; IVR: immersive VR; non-IVR: non-immersive VR; HY: Hoehn and Yeahr; MMSE: Mini-Mental State Examination; PT: physiotherapy; SIBT: Sensory Integration Balance Training.

### VR systems

Ranging from simple commercial gaming devices to highly sophisticated technologies focused on goal-directed tasks, the variety of VR displays already studied in clinical trials highlights its versatility as a rehabilitation tool, with several showing benefits in PD treatment. However, there is no evidence that sophisticated technologies are superior to commercial gaming devices, or vice versa, as comparative studies between VR systems are very few.^34,35^

Nintendo Wii™ and Xbox Kinect™ are the most frequently studied commercial gaming devices in PD rehabilitation using VR. Games from console repertoires are often selected based on the rehabilitation goals, to enable comparisons with similar exercises or conventional physiotherapy. More often than not, the rationale for choosing those games is well explained in the intervention protocol, which is normally done by experienced physiotherapists in the field,^35–38^ and occasionally by a neuropsychologist regarding their cognitive demands.^35^ Other studies^37,39–42^ base their selections in a preliminary research from Mendes et al.,^36^ which investigated pwPD's ability to learn, retain and transfer new motor skills after training with Nintendo Wii™. Importantly, while pwPD were able to improve performance with practice and to retain the benefit after training, they showed learning deficits, particularly when decision-making, attention and working memory were on task.

Nintendo Wii™ and Xbox Kinect™ have been compared in a quasi-experimental study^35^ and in a network meta-analysis.^43^ Despite limitations in methodology, both studies suggest that Nintendo Wii™ might be better suited for pwPD than Xbox Kinect™ because it uses a simpler interface, with less distracting graphics, and it provides a balance board platform that seems more sensitive to detect body transfers of the center of gravity during balance training than the whole-body motion capture technology from Xbox Kinect™. However, one advantage of the Kinect-based system over Nintendo Wii™ is that it allows to run games developed by other companies on its interface. Thus, a commercially available and low-cost display can deliver games specifically designed for rehabilitation targeting specific clinical outcomes.^44–46^

Regarding VR systems specifically designed for rehabilitation, they can be further categorized into those developed for the rehabilitation of several diseases, targeting impairments that are common between neurological diseases^47–51^ and those specially designed for pwPD.^52–55^ Furthermore, VR platforms have been used in various ways within training programs. Sometimes to augment the experience from traditional training methods^51,55,56^ and other times as a standalone intervention.^49,57^ In either case, specific VR systems take advantage of the technology's potentials for the benefit of rehabilitation by promoting highly challenging motor-cognitive interventions adapted to pwPD's specific needs. In fact, these VR devices often have the ability to assess individuals while training and generate data about performance which helps therapists better monitor training and adapt accordingly.^47,51,55,58^

While few studies have compared these two types of VR systems, a subgroup analysis of a recent meta-analysis suggest that rehabilitation-specific systems might be more effective than nonspecific VR systems in improving balance outcomes in PD.^59^ By contrast, one study by Zimmermann and colleagues showed that 4 weeks of nonspecific training with Nintendo Wii™ was slightly superior in improving attention than the group performing CogniPlus™, a task-specific cognitive training program.^60^ Although it seems contradictory, this may suggest that delivering exercises that encompass an interplay of motor and cognitive tasks, such as the case of exergames, might be of greater benefit for pwPD.^61^

VR systems have also been categorized according to the level of immersion in non-immersive, semi-immersive and fully immersive VR systems.^62^ Yet, it is not known if this categorization is relevant in therapeutic interventions. The sense of presence, the illusory feeling of being surrounded by the virtual environment,^31^ depends not only on technical features but also on personal and contextual factors.^63^ Therefore, the same VR system might induce different degrees of sense of presence and influence different behaviors.^20^ Interestingly, one experimental study compared immersive to non-immersive VR training, both targeting hand dexterity in pwPD, showing that both technologies improved fine motor skills of the upper limb without statistical differences between groups.^34^ The authors suggest that task specificity may outweigh the level of immersion in influencing outcomes, although patients were more interested in the immersive VR group. Most studies to date have investigated the effects of non-immersive VR systems in PD outcomes, while studies in fully immersive VR are mainly pilot or feasibility studies with promising results.^48,64–66^

### Settings

The possibility of delivering home-based interventions is one of the attractive features of VR, aiming at reducing the frequency patients need to go to a rehabilitation center, facilitating access to exercise to those living distant and motivating patients to adhere to long-term rehabilitation programs. Although the majority of studies are conducted in-clinic with the supervision of a therapist, some evidence exists of VR interventions delivered at home with remote supervision.

A home-based intervention with Nintendo Wii™ for balance training was compared to a sensory integration balance training (SIBT) delivered in-clinic, in a multicenter RCT.^41^ Exergames were chosen to practice weight shifting, symmetric foot stepping, and controlled movements near the limits of stability. Participants were remotely supervised by videocall (one therapist per two patients) and caregivers were to be present. This training protocol showed high adherence, no adverse events (AE) were reported, and participants significantly improved in static and dynamic postural control when compared to the in-clinic SIBT training. Even though no AE were reported, the authors did not comment on the risk of falling during the use of Nintendo Wii™ at home, particularly in patients with postural instability. Furthermore, while showing promise for the application of this remotely supervised training program, the need for caregiver assistance may limit applicability to all patients.

Two other specifically designed VR systems were investigated as home-based interventions in pwPD with no cognitive impairment.^46,53^ In both RCT sessions were unsupervised, three home visits were performed to ensure correct use, safety and monitor progression, in addition to frequent phone calls. One developed two exergames focusing on coordinated movements of the upper limbs,^53^ and the other a step training platform with cognitive challenges.^46^ While both studies failed to show efficacy, Song et al. suggested a trend towards a benefit in participants with a milder disease severity, whereas participants with more severe disease had a trend towards negative effects in several outcomes, thus indicating that a home-based intervention may be less suitable for this group of pwPD, that may require more supervision.^46^ Regarding the occurrence of AE, eight participants had an exacerbation of a pre-existing pain, considered unrelated to the stepping exercise and one non-injurious fall happened during the exergame, but the authors did not explore this event further.^46^

Another home-based intervention was the Park-in-Shape trial.^55^ This study used cycling in a stationary trainer enhanced by gaming elements and compared it to a stretching training program, also delivered at home. The main objective of this high-quality double-blind RCT was to explore the effect of a high-intensity aerobic exercise in pwPD with mild disease severity. Therefore, although the role of the virtual environment cannot be isolated, this training program coupled with a motivational app allowed an exergaming experience and successfully motivated participants to engage in this 6-month intervention, thus highlighting one of the main strengths of VR systems, which is to increase adherence to prolonged exercise programs. This home-based intervention was considered safe.

Taken together, these studies show that a home-based intervention can be feasible and safe in pwPD but special considerations have to be made regarding the type of intervention and the risk of falling (balance training vs. aerobic exercise in a stationary bicycle), and the appropriate population (pwPD with mild vs. severe disease). In addition, remote supervision is an important common element in these studies to give personalized feedback, keep patients motivated and ensure adherence to training programs. As shown in another study investigating the effect of a home-based completely unsupervised cognitive intervention, pwPD tend to lose interest after several training sessions, especially if games are not varied and if they do not receive frequent feedback from investigators or therapists.^67^

Although not directly explored by the above mentioned studies, a further concern regarding a home-based unsupervised intervention is the risk of musculoskeletal injuries related to compensatory movements by prioritizing game scores over quality of movement.^12,53^ Thus, to prevent complications and make sure that training is always optimized to pwPD's needs, a home-based VR program should probably be complemented periodic in-clinic follow-up.^68^

### Types of exercises and training protocols

As mentioned earlier, although not specifically designed for rehabilitation, games from commercial devices are often selected to address pwPD's impairments. Hence, functionally meaningful tasks are frequently incorporated into the intervention, comprising one or more of the five core areas recommended by the European Guidelines of Physiotherapy.^10^ Some of the involved exercises include full-body motion,^37,69,70^ endurance,^69–71^ strengthening,^42,70,72,73^ stationary gait,^35,39,40,71,74,75^ and static and dynamic balance.^35,37–42,70,72–76^ In addition, the cognitive tasks are commonly recognized within exergames, which may include divided and sustained attention,^35,39–41,72^ planning,^35,38,39,41,76^ and dual tasking.^38,41,76^ Overall, these data suggest that commercialized games can offer a variety of motor and cognitive training and may be adjustable towards rehabilitation goals. For instance, sports games offer boxing, tennis, bowling, kicking and running without the need to go to each of these sports’ places and can meet individuals’ preferences.^37,71,74^ Noteworthy, these scenarios are presented with rewards related to game achievements, visual cues in the form of an avatar, and auditory and proprioceptive stimulus that further engages players for longer periods, thus enhancing the training experience.

Conventional physiotherapy,^40,72,74^ treadmill training,^71^ bicycle training,^37,70,75^ functional gait training,^70,71^ and balance training^39,41,42^ are some of the activities that these VR systems have been compared to, often with equivalent training protocols.^39,40,42,72,74^ Although the variety of games allows to achieve a wide range of therapeutic goals, some studies have incorporated the commercially available VR systems into a program that includes physiotherapy or conventional exercises,^37–39,74^ or even treadmill training,^72^ with VR being used as a complement to conventional rehabilitation. In fact, Santos et al. demonstrated that a combination of VR with conventional physiotherapy results in a larger magnitude of effect compared to using either therapy alone in improving functional balance.^74^ Similarly, Kashif and colleagues combined exergame and motor imagery techniques with physiotherapy resulting in greater improvements than physiotherapy alone in motor severity and balance, with retention effects after one month.^37^ Thus, these studies give some evidence to support the incorporation of VR into a multicomponent exercise program with physiotherapy.^10,77^

Regarding systems specifically designed for rehabilitation, goal-based training is more frequently applied to meet a specific impairment. This training is often combined with gaming elements which offers a challenge in both motor and cognitive pathways and increase motivation.^44,47,52^ Therefore, VR system can be used to augment feedback of a traditional exercise, such as treadmill training^48,51,56,78,79^ or aerobic training,^55^ or even as an intervention in itself. In this case, the VR training might address relevant functional movements, such as balance and postural control,^44,47,54,58^ dynamic balance exercises,^44,49,58,80,81^ high amplitude and fast movements,^52^ leg amplitude,^45^ arm and leg coordination,^44^ and upper limb dexterity.^34,53^ Cognitive training with VR has been delivered as well, as a standalone intervention^50,60,67^ or as part of the VR enhanced feedback, eliciting attentional demand,^46,52,56,58,78^ planning,^44,56^ or dual tasking.^44,48,49,54,56,78^

In contrast with gaming consoles, specific VR systems are less frequently integrated into a multicomponent program. Instead, VR is usually compared to similar exercises or conventional physiotherapy as a sole intervention.^44,46,49,50,53,80,81^ Studies investigating devices developed for rehabilitation may be more focused on proving their effectiveness and isolating their added value for a specific impairment.

### Exercise and neurorehabilitation principles in VR interventions in PD

The positive impact of exercise ranges from physical capacity and symptom improvement to neuroplastic changes, once principles of motor learning and exercise-induced neuroplasticity are targeted.^10,11,82^ As demonstrated in Table 1, both principles of exercise prescription and experience-induced neuroplasticity were present in specific and nonspecific VR studies. Their mention in studies usually aim to explicit the features that made the VR intervention more or less effective and to formulate the arguments for its eventual use in clinical practice.

In studies with nonspecific VR systems, the most common exercise elements included external feedback or cues through multisensory stimuli,^35,37–42,69–72,75,76^ knowledge about performance,^37,38,41,42,71^ and a variety of cognitive challenges.^35,39–42,70–72,76^ A broader range of principles was also identified, such as task specificity,^38,74^ intensity,^38,42,70–72,75^ increase repetitions of movement,^37,39,41,71,72^ progression in difficulty and complexity,^35,38,41,42,69–72^ dual task,^38,41,76^ as well as motivational aspects^39,41,42,69,75^ like awards for the achievements held by the exercise.

While the relationship between the presence of these principles and the effectiveness of VR systems is not possible to establish by analyzing individual studies, it can give preliminary insights. Comparing a nonspecific VR system to similar exercises or conventional physiotherapy, two RCT demonstrated greater improvements in functional balance measures in participants training with a gaming console,^40,41^ where multisensory stimuli, implicit feedback, cognitive engagement and motivation were pointed as possibly associated to the positive results. Additionally, another study investigating the effect of Nintendo Wii™ in clinical and neurobiological outcomes showed improvements in cognition, as measured by MoCA test, visual memory and verbal fluency tasks, when compared to similar conventional exercises.^42^ Using resting-state functional MRI (rs-fMRI), a tool to evaluate functionally related brain regions at rest, the authors found increased precuneus activity following VR training.^42^ As the precuneus functional connectivity with the motor system is decreased in PD, the authors hypothesized that this result represents a normalization of connections within the default mode network, in parallel with improvements in cognitive function and probably related to the enhanced visual and auditory feedback, implicit feedback and motivation provided by the VR system.^42^ Although the presence of these elements seem consistent across positive studies, they do not necessarily guarantee an effective nonspecific VR intervention.^39,70,71,74^

In studies involving specific VR systems, the most commonly reported principles related to VR and exercise were progressive difficulty,^44–46,49–53,56,58,67,78,80,81^ external cueing,^44,47,49,50,52,55,56,58,78,80,81^ implicit feedback,^44,46,50,53–56,78,80,81^ and task specificity.^34,44,48,51,53,56,58,78,80,81^ Also common were intensity,^44,49,54,55,78^ progression in complexity,^44,52,54,56,80^ repetitions of movement,^44,45,52,78,81^ explicit feedback,^45,46,49,50,54,56,81^ dual task and other cognitive challenges,^44,46,48,49,51,52,54,56,58,78,81^ and motivation, interest or reward.^34,45,50,52,53,55,58,67,81^ Interestingly, studies in VR specific systems also refer to the missing elements that could justify less positive results. For example, Allen et al.^53^ used a platform focusing on coordinated movements of the upper limbs, applying only two exergames throughout the 36 sessions. Although the VR system was considered feasible and acceptable, participants manifested a preference for more variety in games, thus emphasizing the importance of varied practice in intervention protocols.^44^ Similarly, the lack of feedback about performance and visual and auditory cueing was identified as the disadvantage of a customized system designed to perform lateral leg movements,^45^ whereas the lack of supervision in a home-based intervention may have influenced low motivation in another study.^67^

When considering studies where VR serves to augment the feedback of a traditional training, the V-TIME project stands out as one of the largest trials, which examined the impact of VR as a motor-cognitive training method on fall rates in a selected population with high risk of falls.^56^ Treadmill training augmented by VR was compared to treadmill training alone. The major clinical finding was a significant decrease in the fall rate in the VR-treadmill treatment group, which was particularly significant in the PD subgroup, for whom the risk of falls reduced by nearly 60% more than treadmill without VR.^56^ The authors propose that the virtual environment trained cognitive pathways involved in obstacle negotiation, such as focused attention, dual-tasking and planning, in addition to the externally-guided rhythmic walking provided by the treadmill, thereby specifically targeting motor and cognitive aspects of fall risk.^56^ Three substudies of the V-TIME project explored the neural correlates associated with these clinical improvements and the plastic effects resulting from training. One study used fMRI to investigate brain activation patterns under motor imagery paradigms.^83^ Following VR-enhanced training, there was a decrease in the activation of frontal areas linked to multitasking, planning, and attention. In contrast, participants in the treadmill training without VR group showed reduced activation in the cerebellum and middle temporal gyrus, areas associated with motor coordination and sensory integration, respectively.^83^ These distinct activation patterns provide insights into the specific nature of the virtual environment, such that by providing motor-cognitive challenges, it improves the efficiency of frontal-striatal circuitry and reduces the need for compensatory cognitive circuits. In another study, these findings were replicated using near infra-red spectroscopy (fNIRS) during actual walking.^84^ Training with the VR system led to a reduction in prefrontal activation in a greater extent than treadmill training alone, particularly in complex walking conditions, giving further evidence of a more efficient recruitment of the prefrontal cortex during dual tasking. In a third study, rs-fMRI was used to compare the modulation of functional networks in a subset of the V-TIME project participants.^85^ Here, there was an improved functional connectivity in the sensorimotor and cerebellar networks after VR-enhanced treadmill training, which was correlated with both usual walking and dual task stride length. In summary, the V-TIME project underscores the role of VR as a goal-directed intervention by virtually placing pwPD in challenging situations demanding divided attention, set shifting, planning, and constant step modification, in a progressive manner, thus facilitating motor learning^24^ and increasing the efficiency of the prefrontal cortex during complex walking conditions.

Overall, these studies suggest that exercise modifies brain activation patterns in an exercise-specific manner and that a multimodal training augmented by VR can offer additional benefits for PD patients in physiotherapy programs. In addition, the positive clinical results and their neural correlates may be linked to VR features that enhance motor learning, including task-specificity, repetition, constant feedback, cognitive engagement, and motivation.

### VR interventions in advanced disease and cognitive impairment

Almost all studies included adults with PD with mild to moderate disease, able to walk unassisted, without cognitive impairment. Therefore, data on the role of VR in more severe disease and cognitively impaired patients is a major gap in the literature of VR interventions, especially for commercial gaming devices.

One study evaluating the efficacy of a non-specified VR system addressing balance and gait training included participants with PD with Hoehn and Yahr (HY) between 2.5 and 4.^73^ Baseline characteristics show that participants were significantly impaired in both groups, as measured by Timed Up and Go test, Berg Balance Scale and Functional Gait assessment, with mean scores representing high risk of falls.^86–88^ Patients significantly improved in these three outcomes following 12 weeks of VR training when compared to a similar traditional exercise. Although the results seem promising for pwPD with postural instability and significant functional impairment, the study lack considerations regarding safety, feasibility, and usability.

In a feasibility study with a VR specific system designed to address freezing of gait and falls, Nuic and colleagues included patients with a mean disease duration of 18 years, HY 3 or 4, with freezing of gait (FOG) or falls despite best medical treatment and subthalamic deep-brain stimulation.^52^ Besides the motor tasks enabling power movements and vertical and forward displacements of lower limbs, this system allowed high number of repetitions, with progressively more challenging tasks, constant multisensory feedback by visual and auditory cues, and high attentional demand. The longitudinal assessment showed significant decrease in FOG severity and number of falls, with kinematic measures confirming better gait performance and balance control.^52^ Besides, the authors considered the VR system feasible and safe, and participants showed good satisfaction and motivation. Therefore, the results from this study together with the one by Song et al.^46^ suggest that patients with more severe disease may require a VR system designed for their specific impairments in a constantly supervised environment.

Cognitive domains have been evaluated in several VR studies as secondary outcomes with few demonstrating improvements.^35,39,42,78^ Nevertheless, only one RCT studied the efficacy of a VR system primarily as a cognitive intervention compared to conventional cognitive training.^50^ In this study, Nirvana BTS demonstrated a significant improvement in all evaluated cognitive domains, including attention, orientation, visual-spatial cognition, language and fluency, memory, and frontal domains, only in the VR group. Interestingly, besides the specific nature of the VR intervention, multisensory stimuli, implicit and explicit feedback, as well as motivation were pointed as additional factors underlying the positive result.

## Clinical and research implications

The use of VR in PD rehabilitation presents promising therapeutic advantages, as indicated by several clinical and neurobiological studies. VR training align principles relevant to neuroplasticity and motor learning in PD, offering goal-oriented tasks, increased repetition and dosage, additional feedback for therapists and patients, and adjustable task difficulty. Multisensory stimuli, in verbal, visual, and proprioceptive forms, provide implicit and explicit feedback, work as external cues to compensate for defective internal feedback, and help patients focusing on the task, increasing cognitive engagement. Given the significant impact of cognitive status on rehabilitation outcomes, strategies emphasizing cognitive engagement are crucial.^10,28^ Moreover, VR has the potential to enhance patient motivation, enjoyment, and interest in intensive task-relevant training programs.

**Figure 1.**
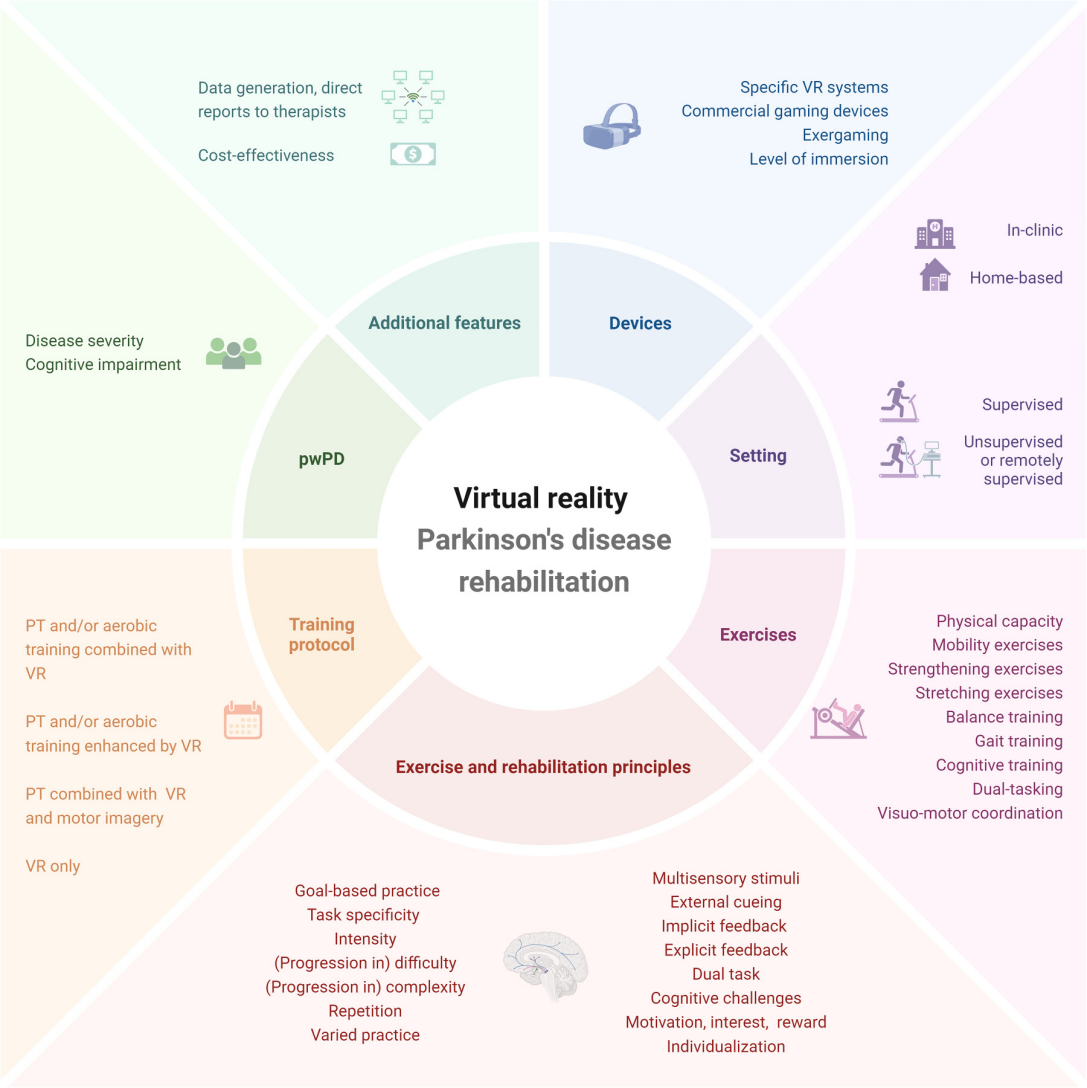
Spectrum of VR-related interventions and its features in Parkinson's disease rehabilitation. VR systems can be divided functionally into commercial gaming devices (e.g., Nintendo Wii or Xbox Kinect) and VR systems specifically designed for rehabilitation. VR can incorporate exergaming, if gaming elements are added to the physical exercise. It can be divided into different levels of immersion. Major research regards VR interventions in-clinic, but it may be suitable for home-based interventions, with remote supervision, in a subset of people with PD. Exercises enabled by VR are broad and versatile towards physiotherapy goals and enhanced by applying principles of exercise prescription and experience-dependent neuroplasticity. Different protocols can be adopted, for example physiotherapy combined with VR or aerobic training enhanced by gaming elements. VR interventions might be suitable to all disease stages and in mild cognitive impairment, provided that goals and exercises are adjusted to disease stage and individuals’ preferences and needs. VR can generate data about performance and report directly to therapists. Finally, some devices may be cost-effective. Created in BioRender. Pimenta Da Silva, D. (2024) BioRender.com/x55c254.

In this review, we described the spectrum of VR systems, VR interventions, training exercises and ways of combining clinical exercise and VR to empower the rehabilitation of PD, which is represented in Figure 1. Some of the advantages of commercial devices are versatility towards treatment goals, constant enhanced feedback provided by visual, auditory and proprioceptive cues, and positive feedback via gaming awards. Therapists can choose through a wide variety of games and set levels of difficulty that can be adapted to each individual. Notably, it can be incorporated into a program with conventional physiotherapy, thus compensating for therapeutic goals not achieved by VR, with evidence showing better results with this multicomponent training solution.^37,74^ While these gaming devices are inexpensive,^35,41,69^ remotely supervised home-based intervention being less expensive than in-clinic,^41^ further research is needed to evaluate its efficacy and safety in this setting. Likewise, there is no evidence regarding its feasibility, efficacy and safety in pwPD with more severe disease and cognitive impairment.

By contrast, specific VR systems are usually designed to integrate goal-based training and neurorehabilitation principles, being its main advantage over nonspecific VR systems. They can be used as a standalone intervention for a specific therapeutic goal, or as an asset within a conventional exercise, thus adding cognitive challenges and enhancing motor learning. In particular, aerobic and treadmill training with gaming elements seem particularly effective.^55,56^ Home-based interventions with these systems may be effective and safe in a subset of patients with mild disease,^46,55^ yet being important to keep a minimal remote supervision to provide positive feedback and keep patients motivated and increase adherence to longer protocols.^55,67^ In addition, pwPD with higher disease stage and mild cognitive impairment can probably benefit from these interventions, when goals and exercise principles are adapted to their disease stage.^10,46,50,52^ Although these specific VR systems tend to be more expensive,^51^ another advantage is the ability to generate an outstanding amount of data about performance and kinematic outcome measures that can be useful for patients and therapists to monitor training and continuously setting goals adjusted to performance.^46,51,55^

Based on current knowledge, we were able to provide some guidance for future research. Larger and higher-quality studies could focus on combined interventions with nonspecific VR systems and conventional physiotherapy, assessing the long-term effects of aerobic and treadmill training with specific VR systems, further exploring home-training with VR in a subset of less disabled patients and designing appropriate devices for patients with more advanced stage and cognitive impairment. A particularly interesting area of research would be to investigate a secondary prevention model with VR, where remotely supervised VR training could alternate with periodic in-clinic supervised exercises to ensure that training is continuously adapted to patients’ specific needs while promoting early and regular physical activity.^68^ Further gaps include defining optimal dosing, frequency and intensity of VR training, as well as systematically reporting adverse events. It would be also interesting to consider appropriate outcomes in VR interventions, as VR systems can provide assessment data and kinematic information which may be more sensitive to detect change in these types of interventions.^89^

## Conclusion

VR is a unique intervention, well suited for rehabilitation because it combines enjoyable exercises with motor-cognitive challenges that can be tailored to individual specific needs and preferences. As a holistic patient-centered approach aiming to maximize the quality of movement and functional independence, physiotherapy can benefit from incorporating a tool like VR to enrich its interventions in the appropriate setting in the multidisciplinary model of care. However, there is still a need for more standardized, theory-driven research. A better understanding of the optimal VR training components and specific outcome measures are essential for advancing the field and maximizing the potential benefits of VR in PD rehabilitation.

## References

[1] Dorsey ER Bloem BR . The Parkinson pandemic-A call to action. JAMA Neurol 2018; 75: 9–10. 29131880 10.1001/jamaneurol.2017.3299

[2] Bloem BR Okun MS Klein C . Parkinson's disease. Lancet 2021; 397: 2284–2303. 33848468 10.1016/S0140-6736(21)00218-X

[3] Fox SH Katzenschlager R Lim SY , et al. International Parkinson and movement disorder society evidence-based medicine review: update on treatments for the motor symptoms of Parkinson's disease. Mov Disord 2018; 33: 1248–1266. 29570866 10.1002/mds.27372

[4] Seppi K Ray Chaudhuri K Coelho M , et al. Update on treatments for nonmotor symptoms of Parkinson's disease-an evidence-based medicine review. Mov Disord 2019; 34: 180–198. 30653247 10.1002/mds.27602 PMC6916382

[5] van der Marck MA Munneke M Mulleners W , et al. Integrated multidisciplinary care in Parkinson's disease: a non-randomised, controlled trial (IMPACT). Lancet Neurol 2013; 12: 947–956. 23988337 10.1016/S1474-4422(13)70196-0

[6] Bloem BR Henderson EJ Dorsey ER , et al. Integrated and patient-centred management of Parkinson's disease: a network model for reshaping chronic neurological care. Lancet Neurol 2020; 19: 623–634. 32464101 10.1016/S1474-4422(20)30064-8 PMC9671491

[7] Radder DLM de Vries NM Riksen NP , et al. Multidisciplinary care for people with Parkinson's disease: the new kids on the block!. Expert Rev Neurother 2019; 19: 145–157. 30570362 10.1080/14737175.2019.1561285

[8] Domingos J Keus SHJ Dean J , et al. The European physiotherapy guideline for Parkinson's disease: implications for neurologists. J Parkinsons Dis 2018; 8: 499–502. 30149464 10.3233/JPD-181383

[9] Radder DLM Ligia Silva de Lima A Domingos J , et al. Physiotherapy in Parkinson's disease: a meta-analysis of present treatment modalities. Neurorehabil Neural Repair 2020; 34: 871–880. 32917125 10.1177/1545968320952799 PMC7564288

[10] Keus SHJ Munneke M Graziano M , et al. European Physiotherapy Guideline for Parkinson's disease. The Netherlands: KNGF/ ParkinsonNet, 2014.

[11] Petzinger GM Fisher BE McEwen S , et al. Exercise-enhanced neuroplasticity targeting motor and cognitive circuitry in Parkinson's disease. Lancet Neurol 2013; 12: 716–726. 23769598 10.1016/S1474-4422(13)70123-6 PMC3690528

[12] Dockx K Bekkers EM Van den Bergh V , et al. Virtual reality for rehabilitation in Parkinson's disease. Cochrane Database Syst Rev 2016; 12: CD010760. 10.1002/14651858.CD010760.pub2 PMC6463967 28000926

[13] Zhang J Luximon Y Pang MYC , et al. Effectiveness of exergaming-based interventions for mobility and balance performance in older adults with Parkinson's disease: systematic review and meta-analysis of randomised controlled trials. Age Ageing 2022; 51: afac175. 10.1093/ageing/afac175 35930726

[14] Yu J Wu J Lu J , et al. Efficacy of virtual reality training on motor performance, activity of daily living, and quality of life in patients with Parkinson's disease: an umbrella review comprising meta-analyses of randomized controlled trials. J Neuroeng Rehabil 2023; 20: 133. 37777748 10.1186/s12984-023-01256-y PMC10544145

[15] Wu J Zhang H Chen Z , et al. Benefits of virtual reality balance training for patients with Parkinson disease: systematic review, meta-analysis, and meta-regression of a randomized controlled trial. JMIR Serious Games 2022; 10: e30882. 10.2196/30882 PMC8924777 35230242

[16] Navarro-Lozano F Kiper P Carmona-Pérez C , et al. Effects of non-immersive virtual reality and video games on walking speed in Parkinson disease: a systematic review and meta-analysis. J Clin Med 2022; 11: 6610. 36431086 10.3390/jcm11226610 PMC9697190

[17] Maier M Rubio Ballester B Duff A , et al. Effect of specific over nonspecific VR-based rehabilitation on poststroke motor recovery: a systematic meta-analysis. Neurorehabil Neural Repair 2019; 33: 112–129. 30700224 10.1177/1545968318820169 PMC6376608

[18] Birckhead B Khalil C Liu X , et al. Recommendations for methodology of virtual reality clinical trials in health care by an international working group: iterative study. JMIR Ment Health 2019; 6: e11973. 10.2196/11973 PMC6374734 30702436

[19] Canning CG Allen NE Nackaerts E , et al. Virtual reality in research and rehabilitation of gait and balance in Parkinson disease. Nat Rev Neurol 2020; 16: 409–425. 32591756 10.1038/s41582-020-0370-2

[20] Perez-Marcos D . Virtual reality experiences, embodiment, videogames and their dimensions in neurorehabilitation. J Neuroeng Rehabil 2018; 15: 113. 30477527 10.1186/s12984-018-0461-0 PMC6258149

[21] Ernst M Folkerts AK Gollan R , et al. Physical exercise for people with Parkinson's disease: a systematic review and network meta-analysis. Cochrane Database Syst Rev 2023; 1: CD013856. 10.1002/14651858.CD013856.pub2 PMC9815433 36602886

[22] Bouca-Machado R Venturelli M Tinazzi M , et al. Treating patients like athletes: sports science applied to Parkinson's disease. Front Neurol 2020; 11: 228. 32300330 10.3389/fneur.2020.00228 PMC7145393

[23] Nieuwboer A Rochester L Muncks L , et al. Motor learning in Parkinson's disease: limitations and potential for rehabilitation. Parkinsonism Relat Disord 2009; 15: S53–S58. 10.1016/S1353-8020(09)70781-3 20083008

[24] Marinelli L Quartarone A Hallett M , et al. The many facets of motor learning and their relevance for Parkinson's disease. Clin Neurophysiol 2017; 128: 1127–1141. 28511125 10.1016/j.clinph.2017.03.042 PMC5486221

[25] Olson M Lockhart TE Lieberman A . Motor learning deficits in Parkinson's disease (PD) and their effect on training response in gait and balance: a narrative review. Front Neurol 2019; 10: 62. 30792688 10.3389/fneur.2019.00062 PMC6374315

[26] Freidle M Thompson WH Albrecht F , et al. Implicit motor sequence learning in people with mild to moderate Parkinson's disease: behavior and related brain function. J Parkinsons Dis 2023; 13: 367–378. 36938739 10.3233/JPD-223480 PMC10200162

[27] Rochester L Burn DJ Woods G , et al. Does auditory rhythmical cueing improve gait in people with Parkinson's disease and cognitive impairment? A feasibility study. Mov Disord 2009; 24: 839–845. 19199354 10.1002/mds.22400

[28] Rochester L Baker K Hetherington V , et al. Evidence for motor learning in Parkinson's disease: acquisition, automaticity and retention of cued gait performance after training with external rhythmical cues. Brain Res 2010; 1319: 103–111. 20064492 10.1016/j.brainres.2010.01.001

[29] Capato TTC de Vries NM IntHout J , et al. Multimodal balance training supported by rhythmical auditory stimuli in Parkinson's disease: a randomized clinical trial. J Parkinsons Dis 2020; 10: 333–346. 31884492 10.3233/JPD-191752 PMC7029328

[30] Ferrazzoli D Ortelli P Madeo G , et al. Basal ganglia and beyond: the interplay between motor and cognitive aspects in Parkinson's disease rehabilitation. Neurosci Biobehav Rev 2018; 90: 294–308. 29733882 10.1016/j.neubiorev.2018.05.007

[31] Bohil CJ Alicea B Biocca FA . Virtual reality in neuroscience research and therapy. Nat Rev Neurosci 2011; 12: 752–762. 22048061 10.1038/nrn3122

[32] Perez-Marcos D Bieler-Aeschlimann M Serino A . Virtual reality as a vehicle to empower motor-cognitive neurorehabilitation. Front Psychol 2018; 9: 2120. 30450069 10.3389/fpsyg.2018.02120 PMC6224455

[33] Maier M Ballester BR Verschure P . Principles of neurorehabilitation after stroke based on motor learning and brain plasticity mechanisms. Front Syst Neurosci 2019; 13: 74. 31920570 10.3389/fnsys.2019.00074 PMC6928101

[34] Cikajlo I Peterlin Potisk K . Advantages of using 3D virtual reality based training in persons with Parkinson's disease: a parallel study. J Neuroeng Rehabil 2019; 16: 119. 31623622 10.1186/s12984-019-0601-1 PMC6798369

[35] Alves MLM Mesquita BS Morais WS , et al. Nintendo Wii™ versus Xbox Kinect™ for assisting people with Parkinson's disease. Percept Mot Skills 2018; 125: 546–565. 29665760 10.1177/0031512518769204

[36] dos Santos Mendes FA Pompeu JE Modenesi Lobo A , et al. Motor learning, retention and transfer after virtual-reality-based training in Parkinson's disease–effect of motor and cognitive demands of games: a longitudinal, controlled clinical study. Physiotherapy 2012; 98: 217–223. 22898578 10.1016/j.physio.2012.06.001

[37] Kashif M Ahmad A Bandpei MAM , et al. Combined effects of virtual reality techniques and motor imagery on balance, motor function and activities of daily living in patients with Parkinson's disease: a randomized controlled trial. BMC Geriatr 2022; 22: 381. 35488213 10.1186/s12877-022-03035-1 PMC9055773

[38] Tollar J Nagy F Kovacs N , et al. A high-intensity multicomponent agility intervention improves Parkinson patients’ clinical and motor symptoms. Arch Phys Med Rehabil 2018; 99: 2478–2484.e2471. 29886075 10.1016/j.apmr.2018.05.007

[39] Pompeu JE Mendes FA Silva KG , et al. Effect of Nintendo Wii™-based motor and cognitive training on activities of daily living in patients with Parkinson's disease: a randomised clinical trial. Physiotherapy 2012; 98: 196–204. 22898575 10.1016/j.physio.2012.06.004

[40] Ribas CG Alves da Silva L Corrêa MR , et al. Effectiveness of exergaming in improving functional balance, fatigue and quality of life in Parkinson's disease: a pilot randomized controlled trial. Parkinsonism Relat Disord 2017; 38: 13–18. 28190675 10.1016/j.parkreldis.2017.02.006

[41] Gandolfi M Geroin C Dimitrova E , et al. Virtual reality telerehabilitation for postural instability in Parkinson's disease: a multicenter, single-blind, randomized, controlled trial. Biomed Res Int 2017; 2017: 7962826. 29333454 10.1155/2017/7962826 PMC5733154

[42] Hajebrahimi F Velioglu HA Bayraktaroglu Z , et al. Clinical evaluation and resting state fMRI analysis of virtual reality based training in Parkinson's disease through a randomized controlled trial. Sci Rep 2022; 12: 8024. 35577874 10.1038/s41598-022-12061-3 PMC9110743

[43] Marotta N Demeco A Indino A , et al. Nintendo Wii(TM) versus Xbox Kinect(TM) for functional locomotion in people with Parkinson's disease: a systematic review and network meta-analysis. Disabil Rehabil 2022; 44: 331–336. 32478581 10.1080/09638288.2020.1768301

[44] Shih MC Wang RY Cheng SJ , et al. Effects of a balance-based exergaming intervention using the Kinect sensor on posture stability in individuals with Parkinson's disease: a single-blinded randomized controlled trial. J Neuroeng Rehabil 2016; 13: 78. 27568011 10.1186/s12984-016-0185-y PMC5002324

[45] Palacios-Navarro G García-Magariño I Ramos-Lorente P . A Kinect-based system for lower limb rehabilitation in Parkinson's disease patients: a pilot study. J Med Syst 2015; 39: 103. 26265237 10.1007/s10916-015-0289-0

[46] Song J Paul SS Caetano MJD , et al. Home-based step training using videogame technology in people with Parkinson's disease: a single-blinded randomised controlled trial. Clin Rehabil 2018; 32: 299–311. 28745063 10.1177/0269215517721593

[47] Pazzaglia C Imbimbo I Tranchita E , et al. Comparison of virtual reality rehabilitation and conventional rehabilitation in Parkinson's disease: a randomised controlled trial. Physiotherapy 2020; 106: 36–42. 32026844 10.1016/j.physio.2019.12.007

[48] Brandín-De la Cruz N Secorro N Calvo S , et al. Immersive virtual reality and antigravity treadmill training for gait rehabilitation in Parkinson's disease: a pilot and feasibility study. Rev Neurol 2020; 71: 447–454. 33319347 10.33588/rn.7112.2020352

[49] van den Heuvel MR Kwakkel G Beek PJ , et al. Effects of augmented visual feedback during balance training in Parkinson's disease: a pilot randomized clinical trial. Parkinsonism Relat Disord 2014; 20: 1352–1358. 25283070 10.1016/j.parkreldis.2014.09.022

[50] Maggio MG De Cola MC Latella D , et al. What about the role of virtual reality in Parkinson disease's cognitive rehabilitation? Preliminary findings from a randomized clinical trial. J Geriatr Psychiatry Neurol 2018; 31: 312–318. 30360679 10.1177/0891988718807973

[51] Calabro RS Naro A Cimino V , et al. Improving motor performance in Parkinson's disease: a preliminary study on the promising use of the computer assisted virtual reality environment (CAREN). Neurol Sci 2020; 41: 933–941. 31858331 10.1007/s10072-019-04194-7

[52] Nuic D Vinti M Karachi C , et al. The feasibility and positive effects of a customised videogame rehabilitation programme for freezing of gait and falls in Parkinson's disease patients: a pilot study. J Neuroeng Rehabil 2018; 15: 31. 29636105 10.1186/s12984-018-0375-x PMC5894136

[53] Allen NE Song J Paul SS , et al. An interactive videogame for arm and hand exercise in people with Parkinson's disease: a randomized controlled trial. Parkinsonism Relat Disord 2017; 41: 66–72. 28528804 10.1016/j.parkreldis.2017.05.011

[54] Maranesi E Casoni E Baldoni R , et al. The effect of non-immersive virtual reality exergames versus traditional physiotherapy in Parkinson's disease older patients: preliminary results from a randomized-controlled trial. Int J Environ Res Public Health 2022; 19: 14818. 36429537 10.3390/ijerph192214818 PMC9690935

[55] van der Kolk NM de Vries NM Kessels RPC , et al. Effectiveness of home-based and remotely supervised aerobic exercise in Parkinson's disease: a double-blind, randomised controlled trial. Lancet Neurol 2019; 18: 998–1008. 31521532 10.1016/S1474-4422(19)30285-6

[56] Mirelman A Rochester L Maidan I , et al. Addition of a non-immersive virtual reality component to treadmill training to reduce fall risk in older adults (V-TIME): a randomised controlled trial. Lancet 2016; 388: 1170–1182. 27524393 10.1016/S0140-6736(16)31325-3

[57] Cheng TC Huang SF Wu SY , et al. Integration of virtual reality into transcranial magnetic stimulation improves cognitive function in patients with Parkinson's disease with cognitive impairment: a proof-of-concept study. J Parkinsons Dis 2022; 12: 723–736. 34897103 10.3233/JPD-212978

[58] Albiol-Pérez S Gil-Gómez JA Muñoz-Tomás MT , et al. The effect of balance training on postural control in patients with Parkinson's disease using a virtual rehabilitation system. Methods Inf Med 2017; 56: 138–144. 28244545 10.3414/ME16-02-0004

[59] Sarasso E Gardoni A Tettamanti A , et al. Virtual reality balance training to improve balance and mobility in Parkinson's disease: a systematic review and meta-analysis. J Neurol 2022; 269: 1873–1888. 34713324 10.1007/s00415-021-10857-3

[60] Zimmermann R Gschwandtner U Benz N , et al. Cognitive training in Parkinson disease: cognition-specific vs nonspecific computer training. Neurology 2014; 82: 1219–1226. 24623840 10.1212/WNL.0000000000000287

[61] Garcia-Agundez A Folkerts AK Konrad R , et al. Recent advances in rehabilitation for Parkinson's disease with exergames: a systematic review. J Neuroeng Rehabil 2019; 16: 17. 30696453 10.1186/s12984-019-0492-1 PMC6352377

[62] Rose T Nam CS Chen KB . Immersion of virtual reality for rehabilitation - review. Appl Ergon 2018; 69: 153–161. 29477323 10.1016/j.apergo.2018.01.009

[63] Hou J Nam Y Peng W , et al. Effects of screen size, viewing angle, and players’ immersion tendencies on game experience. Comput Hum Behav 2012; 28: 617–623.

[64] Campo-Prieto P Cancela-Carral JM Rodriguez-Fuentes G . Wearable immersive virtual reality device for promoting physical activity in Parkinson's disease patients. Sensors (Basel) 2022; 22: 3302. 35590992 10.3390/s22093302 PMC9104114

[65] Ma HI Hwang WJ Fang JJ , et al. Effects of virtual reality training on functional reaching movements in people with Parkinson's disease: a randomized controlled pilot trial. Clin Rehabil 2011; 25: 892–902. 21632652 10.1177/0269215511406757

[66] Kim A Darakjian N Finley JM . Walking in fully immersive virtual environments: an evaluation of potential adverse effects in older adults and individuals with Parkinson's disease. J Neuroeng Rehabil 2017; 14: 16. 28222783 10.1186/s12984-017-0225-2 PMC5320768

[67] van de Weijer SCF Duits AA Bloem BR , et al. Feasibility of a cognitive training game in Parkinson's disease: the randomized Parkin'Play study. Eur Neurol 2020; 83: 426–432. 32756067 10.1159/000509685 PMC7592931

[68] Ellis TD Colon-Semenza C DeAngelis TR , et al. Evidence for early and regular physical therapy and exercise in Parkinson's disease. Semin Neurol 2021; 41: 189–205. 33742432 10.1055/s-0041-1725133 PMC8678920

[69] Herz NB Mehta SH Sethi KD , et al. Nintendo Wii rehabilitation (“Wii-hab”) provides benefits in Parkinson's disease. Parkinsonism Relat Disord 2013; 19: 1039–1042. 23968649 10.1016/j.parkreldis.2013.07.014

[70] Ferraz DD Trippo KV Duarte GP , et al. The effects of functional training, bicycle exercise, and exergaming on walking capacity of elderly patients with Parkinson disease: a pilot randomized controlled single-blinded trial. Arch Phys Med Rehabil 2018; 99: 826–833. 29337023 10.1016/j.apmr.2017.12.014

[71] de Melo GEL Kleiner AFR Lopes JBP , et al. Effect of virtual reality training on walking distance and physical fitness in individuals with Parkinson's disease. Neurotehabilitation 2018; 42: 473–480. 10.3233/NRE-172355 29660956

[72] Liao YY Yang YR Cheng SJ , et al. Virtual reality-based training to improve obstacle-crossing performance and dynamic balance in patients with Parkinson's disease. Neurorehabil Neural Repair 2015; 29: 658–667. 25539782 10.1177/1545968314562111

[73] Feng H Li C Liu J , et al. Virtual reality rehabilitation versus conventional physical therapy for improving balance and gait in Parkinson's disease patients: a randomized controlled trial. Med Sci Monit 2019; 25: 4186–4192. 31165721 10.12659/MSM.916455 PMC6563647

[74] Santos P Machado T Santos L , et al. Efficacy of the Nintendo Wii combination with conventional exercises in the rehabilitation of individuals with Parkinson's disease: a randomized clinical trial. Neurorehabilitation 2019; 45: 255–263. 31498138 10.3233/NRE-192771

[75] Tollár J Nagy F Hortobágyi T . Vastly different exercise programs similarly improve parkinsonian symptoms: a randomized clinical trial. Gerontology 2019; 65: 120–127. 30368495 10.1159/000493127

[76] Pompeu JE Arduini LA Botelho AR , et al. Feasibility, safety and outcomes of playing Kinect Adventures!™ for people with Parkinson's disease: a pilot study. Physiotherapy 2014; 100: 162–168. 24703891 10.1016/j.physio.2013.10.003

[77] Bouca-Machado R Rosario A Caldeira D , et al. Physical activity, exercise, and physiotherapy in Parkinson's disease: defining the concepts. Mov Disord Clin Pract 2020; 7: 7–15. 31970204 10.1002/mdc3.12849 PMC6962680

[78] Mirelman A Maidan I Herman T , et al. Virtual reality for gait training: can it induce motor learning to enhance complex walking and reduce fall risk in patients with Parkinson's disease? J Gerontol A Biol Sci Med Sci 2011; 66: 234–240. 21106702 10.1093/gerona/glq201

[79] Bekkers EMJ Mirelman A Alcock L , et al. Do patients with Parkinson's disease with freezing of gait respond differently than those without to treadmill training augmented by virtual reality? Neurorehabil Neural Repair 2020; 34: 440–449. 32202203 10.1177/1545968320912756

[80] Yen CY Lin KH Hu MH , et al. Effects of virtual reality-augmented balance training on sensory organization and attentional demand for postural control in people with Parkinson disease: a randomized controlled trial. Phys Ther 2011; 91: 862–874. 21474638 10.2522/ptj.20100050

[81] Yang WC Wang HK Wu RM , et al. Home-based virtual reality balance training and conventional balance training in Parkinson's disease: a randomized controlled trial. J Formos Med Assoc 2016; 115: 734–743. 26279172 10.1016/j.jfma.2015.07.012

[82] Abbruzzese G Marchese R Avanzino L , et al. Rehabilitation for Parkinson's disease: current outlook and future challenges. Parkinsonism Relat Disord 2016; 22: S60–S64. 10.1016/j.parkreldis.2015.09.005 26360239

[83] Maidan I Rosenberg-Katz K Jacob Y , et al. Disparate effects of training on brain activation in Parkinson disease. Neurology 2017; 89: 1804–1810. 28954877 10.1212/WNL.0000000000004576

[84] Maidan I Nieuwhof F Bernad-Elazari H , et al. Evidence for differential effects of 2 forms of exercise on prefrontal plasticity during walking in Parkinson's disease. Neurorehabil Neural Repair 2018; 32: 200–208. 29546797 10.1177/1545968318763750

[85] Droby A Maidan I Jacob Y , et al. Distinct effects of motor training on resting-state functional networks of the brain in Parkinson's disease. Neurorehabil Neural Repair 2020; 34: 795–803. 32684069 10.1177/1545968320940985

[86] Schlenstedt C Brombacher S Hartwigsen G , et al. Comparison of the fullerton advanced balance scale, Mini-BESTest, and berg balance scale to predict falls in Parkinson disease. Phys Ther 2016; 96: 494–501. 26381806 10.2522/ptj.20150249

[87] Yang Y Wang Y Zhou Y , et al. Validity of the functional gait assessment in patients with Parkinson disease: construct, concurrent, and predictive validity. Phys Ther 2014; 94: 392–400. 24158644 10.2522/ptj.20130019

[88] Bouca-Machado R Duarte GS Patriarca M , et al. Measurement instruments to assess functional mobility in Parkinson's disease: a systematic review. Mov Disord Clin Pract 2020; 7: 129–139. 32071930 10.1002/mdc3.12874 PMC7011644

[89] Bouca-Machado R Branco D Fonseca G , et al. Kinematic and clinical outcomes to evaluate the efficacy of a multidisciplinary intervention on functional mobility in Parkinson's disease. Front Neurol 2021; 12: 637620. 33833729 10.3389/fneur.2021.637620 PMC8021905

